# Echocardiographic Assessment of Pulmonary Arterial Hypertension After Complete Atrioventricular Septal Defect Repair

**DOI:** 10.1111/echo.70469

**Published:** 2026-04-14

**Authors:** Hannes Sallmon, Martin Koestenberger

**Affiliations:** ^1^ Division of Pediatric Cardiology Department of Pediatrics Medical University of Graz Graz Austria

**Keywords:** AVSD, CAVC, eccentricity index, echocardiography, pulmonary vascular disease

1

Pulmonary arterial hypertension (PAH) is a common finding after anatomic repair of complete atrioventricular septal defects (AVSD, 5%–10 %) [1]. The etiology of postoperative PAH in those patients is multifactorial and includes genetic predisposition (such as in trisomy 21), pre‐operative shunt volumes and duration, and postoperative residual lesions. The situation is further complicated in patients with an additional post‐capillary component as seen in those with (severely) impaired left ventricular function, left atrioventricular valve (LAVV) stenosis, or severe LAVV regurgitation. Concomitant defects or complications such as bundle branch block, pacemaker dependency, pulmonary vein stenosis, or pulmonary pathologies, among others, may add to the complexity of pulmonary vascular disease encountered in postoperative AVSD patients.

While comprehensive cardiac catheterization remains the gold standard to adequately assess postoperative hemodynamics [2], there is a need for noninvasive screening tools for patients at risk for postoperative PAH [2, 3]. This is especially true in pediatric PAH patients when invasive hemodynamic assessments carry a higher risk for potential complications. Therefore, validated echocardiographic screening variables are highly warranted, as they can be (repeatedly) obtained at the bedside, are noninvasive, and potentially useful for long‐term management of postoperative PAH patients. However, an ‘ideal’ screening parameter for pediatric postoperative PAH should fulfill several requirements:

1 Clear cut‐off values with a high sensitivity and acceptable specificity
2 Should be validated in the cohort of interest against the gold standard (cardiac catheterization)
3 Should be easy to assess in different cardiac postoperative anatomies (even in “bad” imaging windows)
4 Should allow for long‐term monitoring of disease progression and response to therapies
5 Should be reproducible and independent of investigator and equipment

In this issue of Echocardiography, Simpkin et al., [4]. Addressed these issues by investigating echocardiographic Eccentricity Index (EI) and its application in screening for postoperative PAH in pediatric AVSD patients. EI is a well‐described echocardiographic predictor of disease severity in children with PAH without congenital heart disease (CHD). EI quantifies the degree of flattening of the intraventricular septum that occurs when the right ventricle faces higher afterload. However, its applicability in postoperative PAH has not been assessed thus far. Theoretically, EI determination in complete AVSD repair may be hampered by the fact that intraventricular septal anatomy and, thus, septal flattening may be altered by surgical patch placement. In addition, pre‐operative shunt burden may lead to right ventricular (RV) remodeling, which potentially affects RV anatomy and EI. Simpkin et al. found, that—despite these theoretical limitations—EI values (systolic and maximum) were consistently increased in patients with postoperative PAH after repair for complete AVSD as compared to those without PAH [4]. Interestingly, this also holds true for patients who exhibited a right bundle branch block. When compared to healthy controls, patients after complete AVSD repair exhibited overall higher EI values independent of their PAH status [4]. The presence of higher EI values even in patients without PAH reflects the possible impact of pre‐operative hemodynamics in complete AVSD and/or the overall higher susceptibility of AVSD patients to (subclinical) pulmonary vascular disease (especially those with trisomy 21) [5]. Importantly, in a second validation cohort who underwent cardiac catheterization, Simpkin et al. showed that systolic EI predicted not only echo‐estimated RV systolic pressures (RVSP) but also invasively determined RVSP and pulmonary vascular resistance index (PVRi) >3 Wu*m [2]. Based on their analyses, the authors suggest a systolic EI threshold of 1.28 to predict elevated RVSP (AUC 0.761) and PVRi (AUC 0.779) [4]. Considering the fact that complete AVSD is a rare condition, this retrospective study cohort of 89 pediatric patients (and 20 patients with invasive hemodynamics) can be considered to be of adequate size. As outlined above, the study addresses the highly relevant problem of adapting established echocardiographic PAH markers to pediatric and, in particular postoperative cohorts [2, 3]. Simpkin et al. chose to investigate EI alongside other markers of RV pressure load and/or function, such as RVSP by tricuspid regurgitation jet, pulmonary arterial acceleration time (PAAT), S/D ratio, and tricuspid annular plane systolic excursion (TAPSE). We agree with the authors that EI is a suitable candidate for a potential PAH screening marker in the postoperative setting of PAH‐CHD for several reasons^2,6,7^: First, EI does not rely on Doppler signals, which can often be incomplete or absent (e.g. tricuspid or pulmonary valve regurgitation jets). Second, EI is obtained from a standard short‐axis view, which is usually recordable even in suboptimal imaging windows. Third, EI has been validated in pediatric PAH and is highly reproducible with low intra‐ or inter‐observer variabilities [6]. Forth, EI has been shown to be predictive of treatment response and outcome in pediatric PAH [7].

In conclusion, Simpkin et al. show that (systolic) EI holds the potential to be a very useful bedside screening tool in pediatric patients with CHD‐PAH. Due to the retrospective and single‐center nature of their study and the limited sample size of patients with invasively obtained hemodynamic data, further prospective validation studies are warranted. Since we know that postoperative hemodynamics change during the first year after complete AVSD repair (e.g., changes in atrioventricular valve regurgitation and gradients) [8, 9], the validity of EI values during long‐term follow‐up and in assessment of treatment response in patients with PAH needs to be further addressed.

Considering the different cut‐off values in patients with AVSD versus controls, the data provided by Simpkin et al. also reminds us that their findings must not be generalized to other postoperative biventricular pathologies with substantial risk of CHD‐PAH (such as d‐transposition of the great arteries, common arterial trunk, biventricular repair of some double‐outlet right ventricle entities, among others). These disease entities all require independent validation studies on suitable echocardiographic markers in order to provide an adequate armamentarium for a multiparametric echocardiographic approach even to complex cases of PAH‐CHD.

## Conflicts of Interest

Drs. Koestenberger and Sallmon indicate no conflicts of interest related to the content of this article.
